# The calcium-dependent protein kinase 1 from *Toxoplasma gondii* as target for structure-based drug design

**DOI:** 10.1017/S0031182017001901

**Published:** 2018-02

**Authors:** EMILY M. CARDEW, CHRISTOPHE L. M. J. VERLINDE, EHMKE POHL

**Affiliations:** 1 Department of Biosciences, Durham University, Lower Mountjoy Durham DH1 3LE, UK; 2 Department of Biochemistry, University of Washington, Seattle, Washington, WA 98195, USA; 3 Department of Chemistry, Durham University, South Road, Durham DH1 3LE, UK; 4 Biophysical Sciences Institute, Durham University, Durham DH1 3LE, UK

**Keywords:** Calcium-dependent protein kinase, CDPK1, *Toxoplasma gondii*, drug design, protein structure

## Abstract

The apicomplexan protozoan parasites include the causative agents of animal and human diseases ranging from malaria (*Plasmodium* spp.) to toxoplasmosis (*Toxoplasma gondii*). The complex life cycle of *T. gondii* is regulated by a unique family of calcium-dependent protein kinases (CDPKs) that have become the target of intensive efforts to develop new therapeutics. In this review, we will summarize structure-based strategies, recent successes and future directions in the pursuit of specific and selective inhibitors of *T. gondii* CDPK1.

## INTRODUCTION

The phylum of Apicomplexa contains approximately 6000 unicellular, eukaryotic parasites including *Plasmodium* spp., the causative agent of Malaria, and *Toxoplasma gondii*, responsible for toxoplasmosis in many important farm animals and humans (Sato, 2011). Morphologically, all members of the apicomplexan family share a distinctive apical complex, together with species dependent apical-localized organelles (McFadden and Yeh, 2017). These parasites employ complex life cycles including both sexual and asexual reproduction. Furthermore, in many cases their life cycles involve multiple hosts. *T. gondii*, first described in 1908 and often regarded as one of the most successful apicomplexan parasites, represents the key model organism of the phylum (Dubey, 2008; Weiss and Dubey, 2009; Szabo and Finney, 2017). Its primary hosts are members of the Felidae (cats) family while all other warm-blooded animals, including humans, are intermediate hosts. It is estimated that up to one third of the human population is infected with *T. gondii* and thus are potential carriers. Although the infection is usually asymptotic in healthy individuals it can cause severe congenital disease during pregnancy (Kaye, 2011), and lead to life-threatening infections in immuno-compromised patients including those suffering from HIV, receiving an organ transplant or undergoing cancer chemotherapy treatment (Flegr *et al.* 2014). Current toxoplasmosis treatment options are limited to a handful of antimicrobials such as sulphonamides, folic acid derivatives and certain macrolide antibiotics. However, these drugs often show limited efficacy and are associated with significant side effects (Alday and Doggett, 2017). Furthermore, there are no treatments available to target tissue cysts, the persistent form in which the parasite evades the host immune system, and to eradicate persistent *T. gondii* infections (Opsteegh *et al.* 2015). Therefore, new drug targets and novel therapies are urgently needed. In addition to high-throughput screening approaches (Norcliffe *et al.* 2014), structure-based methods in close combination with medicinal chemistry and biophysical and biological validation have become powerful tools in the search of new drugs and treatments (Hunter, 2009; Verlinde *et al.* 2009; Groftehauge *et al.* 2015; Hol, 2015; Muller, 2017).

## THE ROLE OF CALCIUM-DEPENDENT PROTEIN KINASES (CDPKs)

In *T. gondii* Ca^2+^-ions play key roles in cell signalling and in pathogen–host interactions including cell invasion, motility of the parasite within the host and differentiation during the parasites complex life cycle (Irvine, 1986; Nagamune *et al.* 2008; Lourido and Moreno, 2015). CDPKs are a family of serine/threonine kinases that are only found in plants and protists including ciliates and apicomplexan parasites. Importantly, these kinases provide the mechanistic link between calcium signalling and motility, differentiation and invasion (Tzen *et al.* 2007; Billker *et al.* 2009). These crucial roles of CDPKs have been proven through a range of knock-out studies in various species and underline the potential of CDPKs as targets for novel therapeutics (Long *et al.* 2016). CDPKs are members of the Calmodulin/Calcium kinase (CaM) family. They all share an N-terminal kinase domain (KD) linked *via* a junctional domain to a series of C-terminal Calcium-binding motifs. In *T. gondii* at least 12 different CDPKs have been putatively identified ranging in size from 507 (CDPK1) to more than 2000 amino acids (CDPK7, CDPK80) (Morlon-Guyot *et al.* 2014). Although there is probably overlap in functionalities, different sub-cellular locations and varying expression levels during the parasites’ life cycle is likely to lead to different biological functions within the CDPK family (Hui *et al.* 2015). The shared sequence identities range from 51% (CDPK1 and CDPK3) (Treeck *et al.* 2014) to lower than 10% (Table 1). These variations in length and sequence support the notion that members of the CDPK family act upon a range of substrates and fulfil different functions in *T. gondii* biology. Recent knock-out studies using CRISPR-Cas9 indicate that CDPK4, CDPK5, CDPK6, CDPK8 and CDPK9, respectively, have no effect on virulence and on normal growth (Wang *et al.* 2016), however, knock-down studies have shown that CDPK7 is crucial for survival due to a critical role in parasite division (Morlon-Guyot *et al.* 2014). More detailed studies have been performed on the smaller family members. CDPK3 with 537 amino acids has been implicated in motility and host cell egress (McCoy *et al.* 2017). CDPK2 (711 amino acids) has been shown to act as key regulator of amylopectin metabolism (Uboldi *et al.* 2015). The loss of CDPK2 results in the build-up of amylum with fatal consequences for *T. gondii* in its chronic stage. Importantly, this family member contains an N-terminal carbohydrate-binding domain that may offer new opportunities for drug design (Uboldi *et al.* 2015). CDPK1, which is mainly located in the cytosol, has been shown to be required for the microneme secretion at the apical complex and parasite proliferation. The molecular mechanism, however, remains elusive (Lourido *et al.* 2010; Child *et al.* 2017). Here we will review strategies and recent results in the discovery, design and potency of inhibitors targeting the KD of CDPK1 from *T. gondii* (*Tg*CDPK1).

**Table 1.** The protein sequence identities between the 12 putative calcium-dependent protein kinases (CDPKs) of *T. gondii*, rounded to the nearest whole number, derived from a multiple sequence alignment (MSA) generated using Clustal Omega (Sievers *et al.* 2011)

	CDPK1	CDPK2	CDPK2A	CDPK2B	CDPK3	CDPK4	CDPK4A	CDPK5	CDPK6	CDPK7	CDPK8	CDPK9
CDPK1		22%	23%	30%	51%	14%	24%	25%	9%	7%	6%	22%
CDPK2	22%		34%	34%	25%	14%	21%	32%	14%	7%	9%	26%
CDPK2A	23%	34%		37%	27%	20%	20%	32%	15%	9%	9%	24%
CDPK2B	30%	34%	37%		33%	16%	25%	36%	13%	8%	8%	26%
CDPK3	51%	25%	27%	33%		14%	25%	30%	12%	7%	7%	25%
CDPK4	14%	14%	20%	16%	14%		14%	15%	17%	11%	9%	14%
CDPK4A	24%	21%	20%	25%	25%	14%		21%	8%	6%	6%	18%
CDPK5	25%	32%	32%	36%	30%	15%	21%		13%	7%	8%	26%
CDPK6	9%	14%	15%	13%	12%	17%	8%	13%		16%	8%	12%
CDPK7	7%	7%	9%	8%	7%	11%	6%	7%	16%		8%	7%
CDPK8	6%	9%	9%	8%	7%	9%	6%	8%	8%	8%		7%
CDPK9	22%	26%	24%	26%	25%	14%	18%	26%	12%	7%	7%	

## ACTIVATION OF *Tg*CDPK1 BY CALCIUM

**Fig. 1.**
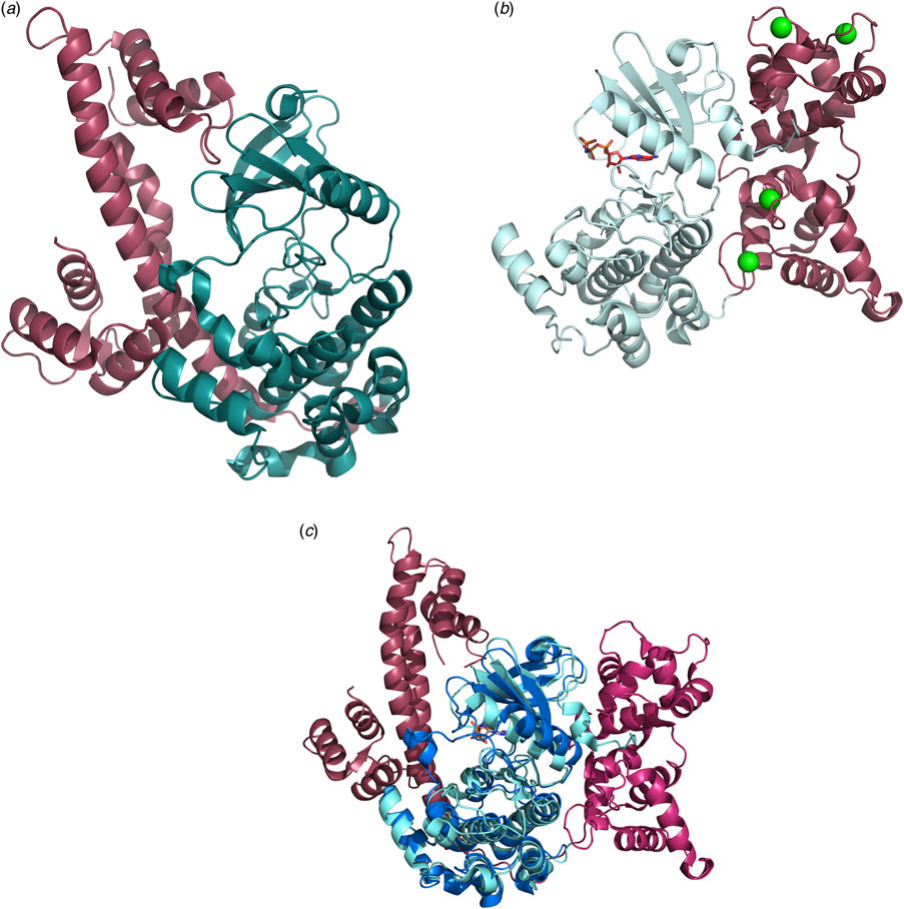
Ribbon representation of the crystal structure of CDPK1 from *T. gondii* with the kinase domain depicted in cyan, the regulatory domain in raspberry red. (a) CDPK1 in its inactive auto-inhibited state (PDB code: 3KU2) (Wernimont *et al.* 2010). (b) CDPK1 in its calcium-bound, activated state with the Ca^2+^-ions shown as green spheres and the non-hydrolysable ligand ANP in stick representation (PDB code: 3HX4) (Wernimont *et al.* 2010). (c) Ribbon diagram of the least-squares superposition of the inactive and active forms of *Tg*CDPK1 with the kinase domains shown in cyan (active) and blue (inactive), the regulatory domain in shades of red, respectively. Only the kinase domain was used to calculate the transformation matrix, which was then applied to the entire protein chain. CDPKs, calcium-dependent protein kinases.

The mechanism of activation and inhibition was unravelled in 2010 when the crystal structures of both the auto-inhibited and the Ca^2+^-activated forms of *Tg*CDPK1 were published (Ojo *et al.* 2010; Wernimont *et al.* 2010). These structures revealed the expected KD in similar overall conformations, however, the Ca^2+^-binding domain (also designated CPDK activating domain or CAD) adopted two vastly different conformations and orientations (Fig. 1). In its inactive state the CAD (shown in rasberry red) adopts an elongated form reminiscent of apo-calmodulin starting with a long helix followed by the first Ca^2+^-binding motifs (EF-hands), which is connected *via* another long helix to the second pair of C-terminal EF-hands (Fig. 1a). The first long helix has been suggested to be responsible for the auto-inhibitory effect by blocking the substrate binding site and providing a basic lysine residue to bind a cluster of conserved acidic residues. However, this may not be the only mechanism of deactivation as it has more recently been shown that removal of the regulatory domain alone does not lead to an active KD (Ingram *et al.* 2015). The CAD domain activated by Ca^2+^-binding appears to be required to maintain the KD in its active conformation. Calcium binding leads to a dramatic rearrangement and refolding of the protein chain (Fig. 1b) (Wernimont *et al.* 2010). The entire regulatory domain is shifted to the other side of the protein hence liberating the active site of the KD as shown in Fig. 1c. In addition, the regulatory calcium-binding domain is collapsed so that the two long helices are no longer arranged in an anti-parallel fashion but are partially unwound and interwoven to form a more globular overall shape. These structural changes are reminiscent to the calcium-bound structure of calmodulin (Kursula, 2014).

## COMPARISON WITH HUMAN KINASES

**Fig. 2.**
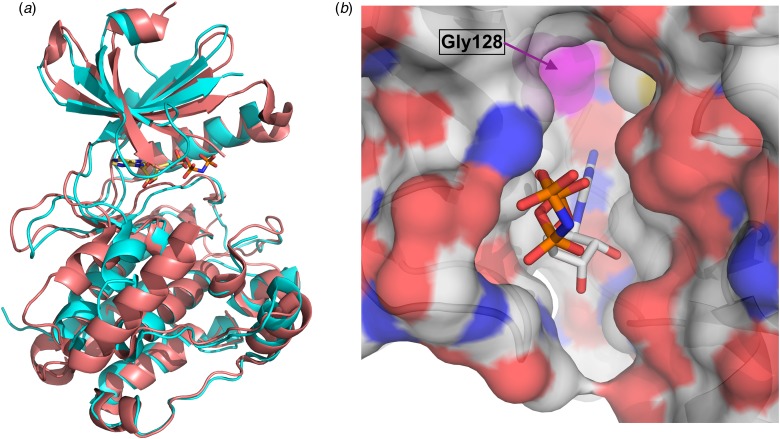
(a) Least squares superposition of the kinase domain of *Tg*CDPK1 (depicted in cyan) in its active form with *Hs*CaMKII bound to an inhibitor (PDB: 2VZ6) (shown in orange) (Rellos *et al.* 2010). The non-hydrolysable ATP analogue bound in *Tg*CDPK1 is presented as ball-and-stick representation to highlight the substrate binding site. (b) Surface representation of *Tg*CDPK1 viewing into the binding pocket with colour coding according to atom type (oxygen in red, nitrogen in blue, carbon in grey). The surface of Gly128 (gatekeeper residue) is shown in magenta at the top of the figure highlighting the additional space in the binding pocket. ATP, adenosine triphosphate; CDPKs, calcium-dependent protein kinases.

Historically, characterising (protozoan) kinases as potential drug targets and developing selective inhibitors has been considered challenging due to the fact that the overall protein fold and the active sites of all kinases are structurally well conserved (Scapin, 2002). The structural similarities of the KD are obvious when comparing the crystal structures of the KD of *Tg*CDPK1 with the Calcium/Calmodulin (CaM) dependent-kinase II from *H. sapiens* (*Hs*CaMKII) (Fig. 2a) (Rellos *et al.* 2010). These two proteins, which share a sequence identity of approximately 42% over 264 residues of the KD, display the same canonical kinase fold and superimpose with an overall root mean square deviation (rmsd) of approximately 1·5 Å. Note that the loop over the adenosine triphosphate (ATP) binding site adopts a very different conformation presumably due to an induced fit of binding of two very different ligands. *Tg*CDPK1 is bound to the ATP analogue ANP (Fig. 2a) while *Hs*CaMKII is bound to a comparatively small inhibitor. More importantly there are significant differences in the ATP binding site, specifically a residue with no side chain (glycine) close to the adenine binding position. This residue, Gly128 is also termed the *gatekeeper* residue. Almost all mammalian kinases possess a large residue, a phenylalanine in *Hs*CaMKII for example, in this position. Hence, CDPK1 feature an enlarged ATP binding site with a hydrophobic pocket that can be exploited for structure-based drug design. This key structural difference in the binding pocket is shown in the surface representation where the ATP-analogue is shown as stick representation (Fig. 2b). The additional space at the end of the pocket below the surface of the gatekeeper residue Gly 128 in magenta is clearly visible.

## DEVELOPMENT OF SPECIFIC *Tg*CDPK1 INHIBITORS

**Fig. 3.**
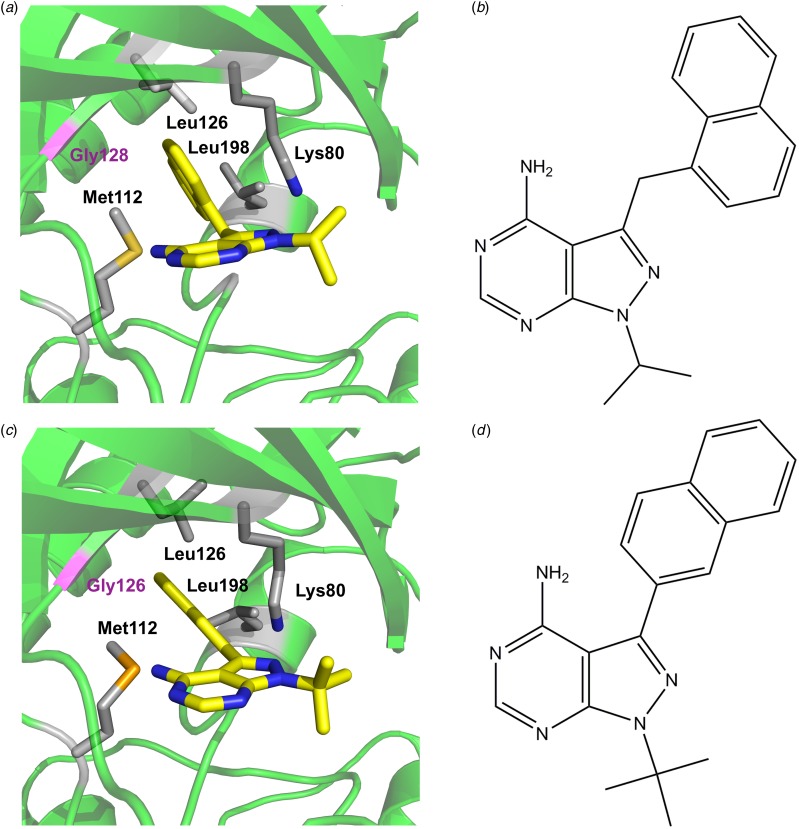
Close-up of BKIs bound to *Tg*CDPK1 in the ATP binding site. The gatekeeper residue Gly128 is depicted in magenta, key hydrophobic residue of the binding site are labelled and shown in grey (a) 1-(1-methylethyl)-3-(naphthalen-1-ylmethyl)-1*H*-pyrazolo[3,4-*d*]pyrimidin-4-amine shown in ball-and-stick representation (PDB: 3i7b), (b) chemical structure of the ligand, (c) 1-tert-butyl-3-naphthalen-2-yl-1*H*-pyrazolo[3,4-*d*]pyrimidin-4-amine (PDB:3i7c) (Ojo *et al.* 2010) and (d) chemical structure of the ligand. ATP, adenosine triphosphate; CDPKs, calcium-dependent protein kinases.

**Fig. 4.**
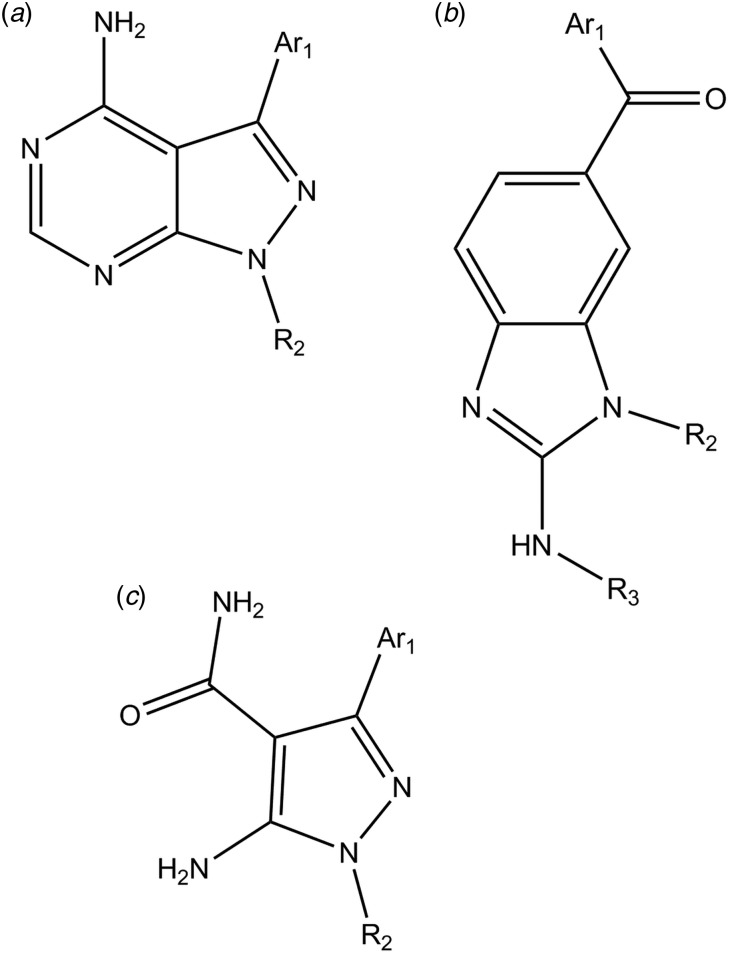
The three different scaffolds for *Tg*CDPK1 inhibitors (a) pyrazolpyrimidines, (b) acylbenzimidazoles and (c) 5-aminopyrazole-4-carboxamide. CDPKs, calcium-dependent protein kinases.

**Fig. 5.**
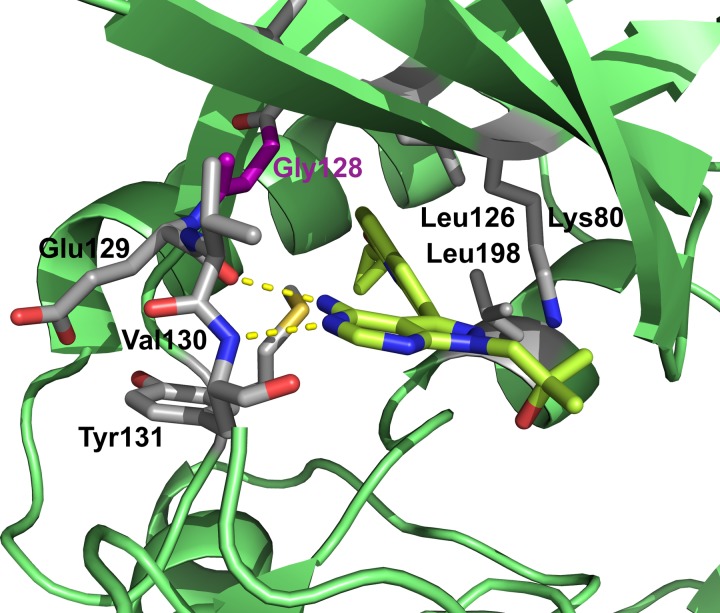
Crystal structure of (1-{4-amino-3-[2-(cyclopropyloxy)quinolin-6-yl]-1*H*-pyrazolo[3,4-*d*]pyrimidin-1-yl}−2-methylpropan-2-ol) shown in stick representation bound to for *Tg*CDPK1 shown in cartoon representation with selected residues depicted in sticks (Vidadala *et al.* 2016). CDPKs, calcium-dependent protein kinases.

Soon after the structural differences between *Tg*CDPK1 and the mammalian homologues were identified, two groups started to develop selective *Tg*CDPK1 inhibitors (Ojo *et al.* 2010; Wernimont *et al.* 2010). Initial compounds were based on known inhibitors previously developed for yeast kinases featuring amino acids with small side chains at the *gatekeeper* position. Importantly, these known kinase inhibitors, termed bumped kinase inhibitors (BKI) have been shown to be inactive against mammalian kinases (Hanke *et al.* 1996). Generally, BKIs are based on the planar pyrazolo[3,4-*d*]pyrimidin-4-amine substituted with a bulky hydrophobic group on the C3 position (Bishop *et al.* 1998). The first example of a BKI with a sub-μmolar IC_50_ is 1-(1-methylethyl)-3-(naphthalen-1-ylmethyl)-1*H*-pyrazolo[3,4-*d*] pyrimidin-4-amine. The co-crystal structure of *Tg*CDPK1 shows that the naphtalen-1-ylmethyl- moiety fills the hydrophobic pocket created by the small gatekeeper residue Gly128 and lined by methionine and leucine residues, and one lysine residue (Fig. 3a and b). The chemically closely related 1-tert-butyl-3-naphthalen-2-yl-1*H*-pyrazolo[3,4-*d*]pyrimidin-4-amine (Fig. 3c and d) adopts a similar conformation with the bulky aromatic substituent at the C3 position occupying the space next to the gatekeeper residue. Critically for the subsequent drug development was the fact that these and related BKIs reduced *T. gondii* proliferation significantly (Ojo *et al.* 2010; Sugi *et al.* 2010). These results sparked extensive medicinal chemistry efforts where a large number of compounds based on the BKI scaffold (4-amino-1*H*-pyrazole[3,4-*d*]pyrimidine) were synthesized and tested. A number of compounds exhibited sub- or low-nanomolar IC_50_ values and high activity in parasite growth models (EC_50_ in the low- and sub-μmolar range) while retaining specificity when compared with mammalian kinases (Lourido *et al.* 2013; Zhang *et al.* 2014; Moine *et al.* 2015). In addition to the pyrazolopyrimidine (PP) scaffolds, acylbenzimidazole and 5-aminopyrzazole-4-carboxamide-based compounds have been shown to have similar properties (Fig. 4) (Zhang *et al.* 2012; Zhang *et al.* 2014; Huang *et al.* 2015). While the initial BKIs showed excellent potency *in vitro* and *in vivo* they also exhibited significant hERG (human Ether-Related Gene) inhibition thus posing potential cardiotoxicity (Doggett *et al.* 2014). Further extensive medicinal chemistry efforts finally led to the current lead *Tg*CDPK1 inhibitor, (1-{4-amino-3-[2-(cyclopropyloxy)quinolin-6-yl]-1*H*-pyrazolo[3,4-*d*]pyrimidin-1-yl}-2-methylpropan-2-ol) that combines high activity and selectivity with favourable pharmacokinetic properties and low hERG activity (Vidadala *et al.* 2016). Note that the compound is bound to the protein *via* H-bonds of the pyrimidin ring to the main chain, while the hydrophobic cyclopropyloxy-quinoline moiety forms a large number of hydrophobic interactions (Fig. 5). Taken together, the structure-based approaches of drug development applied to *Tg*CDPK1 has led to three different series of compounds with high inhibitory activity, good pharmacokinetic parameters and promising efficacy in murine models.

## CDPK1 INHIBITORS FOR RELATED PARASITES

Based on the success of developing specific *Tg*CDPK1 inhibitors, recent work has branched out towards related apicomplexan parasites. For example, *Neospora caninum*, a cyst-forming parasite closely related to *T. gondii* represents the leading cause of abortion in cattle. This parasite expresses a CDPK1 with 96% sequence identity to *Tg*CDPK1 where all residues in the active side are conserved, bar one conservative variation from phenylalanine to tyrosine (Ojo *et al.* 2014). Consequently, the crystal structures of *Tg*CDPK1 and *Nc*CDPK1 show very similar overall structures (root mean square deviations (rmsd) on C-alpha atoms 0·5 Å) and the same binding mode of a BKI. Importantly, a number of BKIs display comparable *in-vivo* activities (Ojo *et al.* 2014; Winzer *et al.* 2015). Members of the *Cryptosporidium* genus are the causative agent of cryptosporidiosis in immune-compromised patients and malnourished children (Shoultz *et al.* 2016). CDPK1 from *C. parvum* Iowa II (*Cp*CDPK1) shares a sequence identity of approximately 41% with *Tg*CDPK1, however, the active site residues including the gatekeeper residue are highly conserved. The screening of BKI libraries resulted in highly active *Cp*CDPK1 inhibitors based on the 5-aminopyrazole-4-carboxamide scaffold with clear potential for drug development (Castellanos-Gonzalez *et al.* 2016). High-throughput screening for *Plasmodium falciparum* CDPK1 (*Pf*CDPK1) inhibitors resulted in five chemical series, including the PP scaffold (Fig. 4a). *Pf*CDPK1 shares a sequence identity of approximately 47% with *Tg*CDPK1 and the gatekeeper residue threonine harbours a slightly larger side chain comparted to glycine. However, this side chain is still relatively small and mutational studies clearly indicated that these inhibitors bind at the same site (Ansell *et al.* 2014). More recent studies with *Pf*CDPK1 inhibitors based on the chemically very similar Imidazopyridazine scaffold appear to show that these compounds also target cyclic GMP dependent kinases as well as Heat Shock Protein 90. These findings question the prospect of *Pf*CDPK1 inhibitors for further drug development (Green *et al.* 2015). Taken together, these recent results show the potential of BKIs for future drug development in Toxoplasma and related parasites but they also illustrate the limitations of transferring detailed structural data to more distantly related proteins.

## FUTURE CHALLENGES

Over the last 5 years there has been significant progress in the development of selective inhibitors of one of the key CDPKs from *T. gondii* achieved by taking advantage of a series of high-resolution crystal structures. While most of the previous research has focused on *T. gondii*, further efforts are currently underway to investigate inhibitors of CDPK1 from Cryptosporidium and *Plasmodium* spp. (Gaji *et al.* 2014; Green *et al.* 2015; Crowther *et al.* 2016). However, further research is required to unravel the biological roles of *Pf*CDPKs and their potential as future drug targets (Kumar *et al.* 2017).

Although the most promising *Tg*CDPK1 inhibitors show high efficacy in murine models, future research is required to increase solubility and bio-availability in order to proceed to clinical trials. Furthermore, current lead compounds only target the ATP binding site of *Tg*CDPK1. However, allosteric kinase inhibitors and modulators have shown enormous potential to target specific kinases and could be further exploited (Fang *et al.* 2013). Additional binding sites in less conserved regions such as the carbohydrate binding site recently discovered in *Tg*CDPK2 can serve as starting points for the development of new inhibitors (Uboldi *et al.* 2015). Clearly, more works needs to be done to understand the role of the other members of the Apicomplexan CDPK family. In this regard, the recent development of CRISPR/Cas9 technology to modify the genes of members of the Apicomplexan family (Shen *et al.* 2014; Vinayak *et al.* 2015) will greatly facilitate the detailed analysis of the biological function of CDPK family members (Long *et al.* 2016; Wang *et al.* 2016).

## References

[ref1] Alday P. H. and Doggett J. S. (2017). Drugs in development for toxoplasmosis: advances, challenges, and current status. Drug Design Development and Therapy 11, 273–293. 10.2147/DDDT.S60973 PMC5279849 28182168

[ref2] Ansell K. H., Jones H. M., Whalley D., Hearn A., Taylor D. L., Patin E. C., Chapman T. M., Osborne S. A., Wallace C., Birchall K., Large J., Bouloc N., Smiljanic-Hurley E., Clough B., Moon R. W., Green J. L. and Holder A. A. (2014). Biochemical and antiparasitic properties of inhibitors of the *Plasmodium falciparum* calcium-dependent protein kinase PfCDPK1. Antimicrobial Agents and Chemotherapy 58, 6032–6043. 25070106 10.1128/AAC.02959-14 PMC4187893

[ref3] Billker O., Lourido S. and Sibley L. D. (2009). Calcium-dependent signaling and kinases in apicomplexan parasites. Cell Host & Microbe 5, 612–622. 19527888 10.1016/j.chom.2009.05.017 PMC2718762

[ref4] Bishop A. C., Shah K., Liu Y., Witucki L., Kung C. and Shokat K. M. (1998). Design of allele-specific inhibitors to probe protein kinase signaling. Current Biology 8, 257–266. 9501066 10.1016/s0960-9822(98)70198-8

[ref5] Castellanos-Gonzalez A., Sparks H., Nava S., Huang W., Zhang Z., Rivas K., Hulverson M. A., Barrett L. K., Ojo K. K., Fan E., Van Voorhis W. C. and White A. C. Jr. (2016). A novel calcium-dependent kinase inhibitor, bumped kinase inhibitor 1517, cures cryptosporidiosis in immunosuppressed mice. Journal of Infectious Diseases 214, 1850–1855. 27738055 10.1093/infdis/jiw481 PMC5142094

[ref6] Child M. A., Garland M., Foe I., Madzelan P., Treeck M., van der Linden W. A., Oresic Bender K., Weerapana E., Wilson M. A., Boothroyd J. C., Reese M. L. and Bogyo M. (2017). Toxoplasma DJ-1 regulates organelle secretion by a direct interaction with calcium-dependent protein kinase 1. MBio 8, e02189-16. 10.1128/mBio.02189-16 PMC5347346 28246362

[ref7] Crowther G. J., Hillesland H. K., Keyloun K. R., Reid M. C., Lafuente-Monasterio M. J., Ghidelli-Disse S., Leonard S. E., He P., Jones J. C., Krahn M. M., Mo J. S., Dasari K. S., Fox A. M., Boesche M., El Bakkouri M., Rivas K. L., Leroy D., Hui R., Drewes G., Maly D. J., Van Voorhis W. C. and Ojo K. K. (2016). Biochemical screening of five protein kinases from *Plasmodium falciparum* against 14 000 cell-active compounds. PLoS ONE 11, e0149996. 26934697 10.1371/journal.pone.0149996 PMC4774911

[ref8] Doggett J. S., Ojo K. K., Fan E., Maly D. J. and Van Voorhis W. C. (2014). Bumped kinase inhibitor 1294 treats established *Toxoplasma gondii* infection. Antimicrobial Agents and Chemotherapy 58, 3547–3549. 24687502 10.1128/AAC.01823-13 PMC4068437

[ref9] Dubey J. P. (2008). The history of *Toxoplasma gondii* – the first 100 years. Journal of Eukaryotic Microbiology 55, 467–475. 19120791 10.1111/j.1550-7408.2008.00345.x

[ref10] Fang Z., Grutter C. and Rauh D. (2013). Strategies for the selective regulation of kinases with allosteric modulators: exploiting exclusive structural features. ACS Chemical Biology 8, 58–70. 23249378 10.1021/cb300663j

[ref11] Flegr J., Prandota J., Sovickova M. and Israili Z. H. (2014). Toxoplasmosis – a global threat. Correlation of latent toxoplasmosis with specific disease burden in a set of 88 countries. PLoS ONE 9, e90203. 24662942 10.1371/journal.pone.0090203 PMC3963851

[ref12] Gaji R. Y., Checkley L., Reese M. L., Ferdig M. T. and Arrizabalaga G. (2014). Expression of the essential kinase *Pf*CDPK1 from *Plasmodium falciparum* in *Toxoplasma gondii* facilitates the discovery of novel antimalarial drugs. Antimicrobial Agents and Chemotherapy 58, 2598–2607. 24550330 10.1128/AAC.02261-13 PMC3993251

[ref13] Green J. L., Moon R. W., Whalley D., Bowyer P. W., Wallace C., Rochani A., Nageshan R. K., Howell S. A., Grainger M., Jones H. M., Ansell K. H., Chapman T. M., Taylor D. L., Osborne S. A., Baker D. A., Tatu U. and Holder A. A. (2015). Imidazopyridazine inhibitors of *Plasmodium falciparum* calcium-dependent protein kinase 1 also target cyclic GMP-dependent protein kinase and heat shock protein 90 to kill the parasite at different stages of intracellular development. Antimicrobial Agents and Chemotherapy 60, 1464–1475. 26711771 10.1128/AAC.01748-15 PMC4775997

[ref14] Groftehauge M. K., Hajizadeh N. R., Swann M. J. and Pohl E. (2015). Protein-ligand interactions investigated by thermal shift assays (TSA) and dual polarization interferometry (DPI). Acta Crystallographica Section D, Biological Crystallography 71, 36–44. 25615858 10.1107/S1399004714016617 PMC4304684

[ref15] Hanke J. H., Gardner J. P., Dow R. L., Changelian P. S., Brissette W. H., Weringer E. J., Pollok B. A. and Connelly P. A. (1996). Discovery of a novel, potent, and Src family-selective tyrosine kinase inhibitor. Study of Lck- and FynT-dependent T cell activation. Journal of Biological Chemistry 271, 695–701. 8557675 10.1074/jbc.271.2.695

[ref16] Hol W. G. (2015). Three-dimensional structures in the design of therapeutics targeting parasitic protozoa: reflections on the past, present and future. Acta Crystallographica F Structural Biology Communications 71, 485–499. 25945701 10.1107/S2053230X15004987 PMC4427157

[ref17] Huang W., Ojo K. K., Zhang Z., Rivas K., Vidadala R. S., Scheele S., DeRocher A. E., Choi R., Hulverson M. A., Barrett L. K., Bruzual I., Siddaramaiah L. K., Kerchner K. M., Kurnick M. D., Freiberg G. M., Kempf D., Hol W. G., Merritt E. A., Neckermann G., de Hostos E. L., Isoherranen N., Maly D. J., Parsons M., Doggett J. S., Van Voorhis W. C. and Fan E. (2015). SAR studies of 5-aminopyrazole-4-carboxamide analogues as potent and selective inhibitors of *Toxoplasma gondii* CDPK1. ACS Medicinal Chemistry Letters 6, 1184–1189. 26693272 10.1021/acsmedchemlett.5b00319 PMC4677665

[ref18] Hui R., El Bakkouri M. and Sibley L. D. (2015). Designing selective inhibitors for calcium-dependent protein kinases in apicomplexans. Trends in Pharmacological Sciences 36, 452–460. 26002073 10.1016/j.tips.2015.04.011 PMC4485940

[ref19] Hunter W. N. (2009). Structure-based ligand design and the promise held for antiprotozoan drug discovery. Journal of Biological Chemistry 284, 11749–11753. 19103598 10.1074/jbc.R800072200 PMC2673241

[ref20] Ingram J. R., Knockenhauer K. E., Markus B. M., Mandelbaum J., Ramek A., Shan Y., Shaw D. E., Schwartz T. U., Ploegh H. L. and Lourido S. (2015). Allosteric activation of apicomplexan calcium-dependent protein kinases. Proceedings of the National Academy of Sciences of the United States of America 112, E4975–E4984. 26305940 10.1073/pnas.1505914112 PMC4568647

[ref21] Irvine R. F. (1986). Calcium transients: mobilization of intracellular Ca^2+^. British Medical Bulletin 42, 369–374. 2820539 10.1093/oxfordjournals.bmb.a072154

[ref22] Kaye A. (2011). Toxoplasmosis: diagnosis, treatment, and prevention in congenitally exposed infants. Journal of Pediatric Health Care 25, 355–364. 22018426 10.1016/j.pedhc.2010.04.008

[ref23] Kumar S., Kumar M., Ekka R., Dvorin J. D., Paul A. S., Madugundu A. K., Gilberger T., Gowda H., Duraisingh M. T., Keshava Prasad T. S. and Sharma P. (2017). *Pf*CDPK1 mediated signaling in erythrocytic stages of *Plasmodium falciparum*. Nature Communications 8, 63. 10.1038/s41467-017-00053-1 PMC5498596 28680058

[ref24] Kursula P. (2014). The many structural faces of calmodulin: a multitasking molecular jackknife. Amino Acids 46, 2295–2304. 25005783 10.1007/s00726-014-1795-y

[ref25] Long S., Wang Q. and Sibley L. D. (2016). Analysis of noncanonical calcium-dependent protein kinases in *Toxoplasma gondii* by targeted gene deletion using CRISPR/Cas9. Infection and Immunity 84, 1262–1273. 26755159 10.1128/IAI.01173-15 PMC4862710

[ref26] Lourido S. and Moreno S. N. (2015). The calcium signaling toolkit of the Apicomplexan parasites *Toxoplasma gondii* and *Plasmodium* spp. Cell Calcium 57, 186–193. 25605521 10.1016/j.ceca.2014.12.010 PMC4428288

[ref27] Lourido S., Shuman J., Zhang C., Shokat K. M., Hui R. and Sibley L. D. (2010). Calcium-dependent protein kinase 1 is an essential regulator of exocytosis in *Toxoplasma*. Nature 465, 359–362. 20485436 10.1038/nature09022 PMC2874977

[ref28] Lourido S., Jeschke G. R., Turk B. E. and Sibley D. (2013). Exploiting the unique ATP-binding pocket of toxoplasma calcium-dependent protein kinase 1 to identify its substrates. ACS Chemical Biology 8, 1155–1162. 23530747 10.1021/cb400115y PMC3691715

[ref29] McCoy J. M., Stewart R. J., Uboldi A. D., Li D., Schroder J., Scott N. E., Papenfuss A. T., Lehane A. M., Foster L. J. and Tonkin C. J. (2017). A forward genetic screen identifies a negative regulator of rapid Ca^2+^-dependent cell egress (MS1) in the intracellular parasite *Toxoplasma gondii*. Journal of Biological Chemistry 292, 7662–7674. 28258212 10.1074/jbc.M117.775114 PMC5418062

[ref30] McFadden G. I. and Yeh E. (2017). The apicoplast: now you see it, now you don't. International Journal for Parasitology 47, 137–144. 27773518 10.1016/j.ijpara.2016.08.005 PMC5406208

[ref31] Moine E., Dimier-Poisson I., Enguehard-Gueiffier C., Logé C., Pénichon M., Moiré N., Delehouzé C., Foll-Josselin B., Ruchaud S., Bach S., Gueiffier A., Debierre-Grockiego F. and Denevault-Sabourin C. (2015). Development of new highly potent imidazo[1,2-*b*]pyridazines targeting *Toxoplasma gondii* calcium-dependent protein kinase 1. European Journal of Medicinal Chemistry 105, 80–105. 26479029 10.1016/j.ejmech.2015.10.004

[ref32] Morlon-Guyot J., Berry L., Chen C. T., Gubbels M. J., Lebrun M. and Daher W. (2014). The *Toxoplasma gondii* calcium-dependent protein kinase 7 is involved in early steps of parasite division and is crucial for parasite survival. Cellular Microbiology 16, 95–114. 24011186 10.1111/cmi.12186 PMC4091637

[ref33] Muller I. (2017). Guidelines for the successful generation of protein-ligand complex crystals. Acta Crystallographica D Structural Biology 73, 79–92. 28177304 10.1107/S2059798316020271 PMC5297911

[ref34] Nagamune K., Moreno S. N., Chini E. N. and Sibley L. D. (2008). Calcium regulation and signaling in apicomplexan parasites. Subcellular Biochemistry 47, 70–81. 18512342 10.1007/978-0-387-78267-6_5

[ref35] Norcliffe J. L., Alvarez-Ruiz E., Martin-Plaza J. J., Steel P. G. and Denny P. W. (2014). The utility of yeast as a tool for cell-based, target-directed high-throughput screening. Parasitology 141, 8–16. 23611102 10.1017/S0031182013000425

[ref36] Ojo K. K., Larson E. T., Keyloun K. R., Castaneda L. J., Derocher A. E., Inampudi K. K., Kim J. E., Arakaki T. L., Murphy R. C., Zhang L., Napuli A. J., Maly D. J., Verlinde C. L., Buckner F. S., Parsons M., Hol W. G., Merritt E. A. and Van Voorhis W. C. (2010). *Toxoplasma gondii* calcium-dependent protein kinase 1 is a target for selective kinase inhibitors. Nature Structural & Molecular Biology 17, 602–607. 10.1038/nsmb.1818 PMC2896873 20436472

[ref37] Ojo K. K., Reid M. C., Kallur Siddaramaiah L., Muller J., Winzer P., Zhang Z., Keyloun K. R., Vidadala R. S., Merritt E. A., Hol W. G., Maly D. J., Fan E., Van Voorhis W. C. and Hemphill A. (2014). *Neospora caninum* calcium-dependent protein kinase 1 is an effective drug target for neosporosis therapy. PLoS ONE 9, e92929. 24681759 10.1371/journal.pone.0092929 PMC3969379

[ref38] Opsteegh M., Kortbeek T. M., Havelaar A. H. and van der Giessen J. W. (2015). Intervention strategies to reduce human *Toxoplasma gondii* disease burden. Clinical Infectious Diseases 60, 101–107. 25225234 10.1093/cid/ciu721

[ref39] Rellos P., Pike A. C., Niesen F. H., Salah E., Lee W. H., von Delft F. and Knapp S. (2010). Structure of the CaMKIIdelta/calmodulin complex reveals the molecular mechanism of CaMKII kinase activation. PLoS Biology 8, e1000426. 20668654 10.1371/journal.pbio.1000426 PMC2910593

[ref40] Sato S. (2011). The apicomplexan plastid and its evolution. Cellular and Molecular Life Sciences 68, 1285–1296. 21380560 10.1007/s00018-011-0646-1 PMC3064897

[ref41] Scapin G. (2002). Structural biology in drug design: selective protein kinase inhibitors. Drug Discovery Today 7, 601–611. 12047871 10.1016/s1359-6446(02)02290-0

[ref42] Shen B., Brown K. M., Lee T. D. and Sibley L. D. (2014). Efficient gene disruption in diverse strains of *Toxoplasma gondii* using CRISPR/CAS9. MBio 5, e01114–e01114. 24825012 10.1128/mBio.01114-14 PMC4030483

[ref43] Shoultz D. A., de Hostos E. L. and Choy R. K. (2016). Addressing cryptosporidium infection among young children in low-income settings: the crucial role of new and existing drugs for reducing morbidity and mortality. PLoS Neglected Tropical Diseases 10, e0004242. 26820408 10.1371/journal.pntd.0004242 PMC4731073

[ref44] Sievers F., Wilm A., Dineen D., Gibson T. J., Karplus K., Li W., Lopez R., McWilliam H., Remmert M., Söding J., Thompson J. D. and Higgins D. G. (2011). Fast, scalable generation of high-quality protein multiple sequence alignments using Clustal Omega. Molecular Systems Biology 7, 539. 21988835 10.1038/msb.2011.75 PMC3261699

[ref45] Sugi T., Kato K., Kobayashi K., Watanabe S., Kurokawa H., Gong H., Pandey K., Takemae H. and Akashi H. (2010). Use of the kinase inhibitor analog 1NM-PP1 reveals a role for *Toxoplasma gondii* CDPK1 in the invasion step. Eukaryotic Cell 9, 667–670. 20173034 10.1128/EC.00351-09 PMC2863409

[ref46] Szabo E. K. and Finney C. A. (2017). *Toxoplasma gondii*: one organism, multiple models. Trends in Parasitology 33, 113–127. 27988095 10.1016/j.pt.2016.11.007

[ref47] Treeck M., Sanders J. L., Gaji R. Y., LaFavers K. A., Child M. A., Arrizabalaga G., Elias J. E. and Boothroyd J. C. (2014). The calcium-dependent protein kinase 3 of toxoplasma influences basal calcium levels and functions beyond egress as revealed by quantitative phosphoproteome analysis. PLoS Pathogens 10, e1004197. 24945436 10.1371/journal.ppat.1004197 PMC4063958

[ref48] Tzen M., Benarous R., Dupouy-Camet J. and Roisin M. P. (2007). A novel *Toxoplasma gondii* calcium-dependent protein kinase. Parasite 14, 141–147. 17645186 10.1051/parasite/2007142141

[ref49] Uboldi A. D., McCoy J. M., Blume M., Gerlic M., Ferguson D. J., Dagley L. F., Beahan C. T., Stapleton D. I., Gooley P. R., Bacic A., Masters S. L., Webb A. I., McConville M. J. and Tonkin C. J. (2015). Regulation of starch stores by a Ca(2+)-dependent protein kinase Is essential for viable cyst development in *Toxoplasma gondii*. Cell Host & Microbe 18, 670–681. 26651943 10.1016/j.chom.2015.11.004

[ref50] Verlinde C. L., Fan E., Shibata S., Zhang Z., Sun Z., Deng W., Ross J., Kim J., Xiao L., Arakaki T. L., Bosch J., Caruthers J. M., Larson E. T., Letrong I., Napuli A., Kelly A., Mueller N., Zucker F., Van Voorhis W. C., Buckner F. S., Merritt E. A. and Hol W. G. (2009). Fragment-based cocktail crystallography by the medical structural genomics of pathogenic protozoa consortium. Current Topics in Medicinal Chemistry 9, 1678–1687. 19929835 10.2174/156802609790102383 PMC2897734

[ref51] Vidadala R. S., Rivas K. L., Ojo K. K., Hulverson M. A., Zambriski J. A., Bruzual I., Schultz T. L., Huang W., Zhang Z., Scheele S., DeRocher A. E., Choi R., Barrett L. K., Siddaramaiah L. K., Hol W. G., Fan E., Merritt E. A., Parsons M., Freiberg G., Marsh K., Kempf D. J., Carruthers V. B., Isoherranen N., Doggett J. S., Van Voorhis W. C. and Maly D. J. (2016). Development of an orally available and central nervous system (CNS) penetrant *Toxoplasma gondii* calcium-dependent protein kinase 1 (*Tg*CDPK1) inhibitor with minimal human ether-a-go-go-related gene (hERG) activity for the treatment of toxoplasmosis. Journal of Medicinal Chemistry 59, 6531–6546. 27309760 10.1021/acs.jmedchem.6b00760 PMC5100899

[ref52] Vinayak S., Pawlowic M. C., Sateriale A., Brooks C. F., Studstill C. J., Bar-Peled Y., Cipriano M. J. and Striepen B. (2015). Genetic modification of the diarrhoeal pathogen *Cryptosporidium parvum*. Nature 523, 477–480. 26176919 10.1038/nature14651 PMC4640681

[ref53] Wang J. L., Huang S. Y., Li T. T., Chen K., Ning H. R. and Zhu X. Q. (2016). Evaluation of the basic functions of six calcium-dependent protein kinases in *Toxoplasma gondii* using CRISPR-Cas9 system. Parasitology Research 115, 697–702. 26499803 10.1007/s00436-015-4791-6

[ref54] Weiss L. M. and Dubey J. P. (2009). Toxoplasmosis: a history of clinical observations. International Journal for Parasitology 39, 895–901. 19217908 10.1016/j.ijpara.2009.02.004 PMC2704023

[ref55] Wernimont A. K., Artz J. D., Finerty P. Jr., Lin Y. H., Amani M., Allali-Hassani A., Senisterra G., Vedadi M., Tempel W., Mackenzie F., Chau I., Lourido S., Sibley L. D. and Hui R. (2010). Structures of apicomplexan calcium-dependent protein kinases reveal mechanism of activation by calcium. Nature Structural & Molecular Biology 17, 596–601. 10.1038/nsmb.1795 PMC3675764 20436473

[ref56] Winzer P., Müller J., Aguado-Martinez A., Rahman M., Balmer V., Manser V., Ortega-Mora L. M., Ojo K. K., Fan E., Maly D. J., Van Voorhis W. C. and Hemphill A. (2015). In vitro and in vivo effects of the bumped kinase inhibitor 1294 in the related cyst-forming apicomplexans *Toxoplasma gondii* and *Neospora caninum*. Antimicrobial Agents and Chemotherapy 59, 6361–6374. 26248379 10.1128/AAC.01236-15 PMC4576114

[ref57] Zhang Z., Ojo K. K., Johnson S. M., Larson E. T., He P., Geiger J. A., Castellanos-Gonzalez A., White A. C. Jr., Parsons M., Merritt E. A., Maly D. J., Verlinde C. L., Van Voorhis W. C. and Fan E. (2012). Benzoylbenzimidazole-based selective inhibitors targeting *Cryptosporidium parvum* and *Toxoplasma gondii* calcium-dependent protein kinase-1. Bioorganic & Medicinal Chemistry Letters 22, 5264–5267. 22795629 10.1016/j.bmcl.2012.06.050 PMC3420979

[ref58] Zhang Z., Ojo K. K., Vidadala R., Huang W., Geiger J. A., Scheele S., Choi R., Reid M. C., Keyloun K. R., Rivas K., Siddaramaiah L. K., Comess K. M., Robinson K. P., Merta P. J., Kifle L., Hol W. G., Parsons M., Merritt E. A., Maly D. J., Verlinde C. L., Van Voorhis W. C. and Fan E. (2014). Potent and selective inhibitors of CDPK1 from *T. gondii* and *C. parvum* based on a 5-aminopyrazole-4-carboxamide scaffold. ACS Medical Chemistry Letters 5, 40–44. 10.1021/ml400315s PMC3908674 24494061

